# Distribution of porcine monocytes in different lymphoid tissues and the lungs during experimental *Actinobacillus pleuropneumoniae* infection and the role of chemokines

**DOI:** 10.1186/1297-9716-44-98

**Published:** 2013-10-17

**Authors:** Petra Ondrackova, Lenka Leva, Zdenka Kucerova, Monika Vicenova, Marketa Mensikova, Martin Faldyna

**Affiliations:** 1 Veterinary Research Institute, Hudcova 70, Brno 621 00, Czech Republic

## Abstract

Monocytes play an essential role in the defense against bacterial pathogens. Bone marrow (BM) and peripheral blood (PB) monocytes in pigs consist of the main “steady-state” subpopulations: CD14^hi^/CD163^-^/SLA-DR^-^ and CD14^low^/CD163^+^/SLA-DR^+^. During inflammation, the subpopulation of “inflammatory” monocytes expressing very high levels of CD163, but lacking the SLA-DR molecule (being CD14^low^/CD163^+^/SLA-DR^-^) appears in the BM and PB and replaces the CD14^low^/CD163^+^/SLA-DR^+^ subpopulation. However, current knowledge of monocyte migration into inflamed tissues in pigs is limited. The aim of the present study was to evaluate the distribution of “inflammatory” CD14^low^/CD163^+^/SLA-DR^-^ monocytes during experimental inflammation induced by *Actinobacillus pleuropneumoniae* (APP) and a possible role for chemokines in attracting “inflammatory” CD14^low^/CD163^+^/SLA-DR^-^ monocytes into the tissues. Monocyte subpopulations were detected by flow cytometry. Chemokines and chemokine receptors were detected by RT-qPCR. The “steady-state” monocytes were found in the BM, PB, spleen and lungs of control pigs. After APP-infection, “inflammatory” monocytes replaced the “steady-state” subpopulation in BM, PB, spleen and moreover, they appeared in an unaffected area, demarcation zone and necrotic area of the lungs and in tracheobronchial lymph nodes. They did not appear in mesenteric lymph nodes. Levels of mRNA for various chemokines with their appropriate receptors were found to be elevated in BM (CCL3-CCR1/CCR5, CCL8-CCR2/CCR5, CCL19-CCR7), necrotic area of the lungs (CCL3-CCR1, CCL5-CCR1/CCR3, CCL11-CCR3, CCL22/CCR4) and tracheobronchial lymph nodes (CCL3-CCR1) and therefore they could play a role in attracting monocytes into inflamed tissues. In conclusion, “inflammatory” monocytes appear in different lymphoid tissues and the lungs after APP infection in pigs. Various chemokines could drive this process.

## Introduction

Monocytes are cells of nonspecific immunity which play an essential role in the defense against bacterial pathogens [1]. Monocytes originate from bone marrow (BM). Monocytes from the BM enter blood circulation and from there they migrate to various tissues. Thereafter, they can undergo further differentiation into macrophages or dendritic cells (DC) during inflammation [2,3]. Moreover, monocytes themselves can infiltrate the site of inflammation and subsequently the draining lymph nodes (LN) where they present the antigen as monocyte-derived dendritic cells [2,4]. Recent findings show that monocytes are also capable of transporting the antigen from the tissue to the LN [5,6] where they efficiently present the antigen to T cells as they are, i.e. without subsequent development into DC [7].

An ambivalent role is attributed to the spleen in monocyte trafficking. Monocytes recruited into the spleen during inflammation play an important role in the control of the infection as they are [8] or they can develop into a specialized subset of DC (the TipDC) which play a crucial role in controlling pathogen burden [3]. The spleen, however, is populated with monocytes, also under steady-state conditions, serving as a rapid source of monocytes in the event of their sudden need [9].

Recruitment of monocytes from the BM to the PB and from PB to inflamed tissues and LN in mice is driven by chemokines, chemokine receptors [10-12], adhesion molecules [13] and other factors [14]. Monocytes are mobilized from the BM to the PB via CCR2-CCL2/CCL7 signaling [15]. The role of CCR2-CCL2 signaling is controversial, while other chemokines and their receptors such as CCL3, CCL4, CCR1 and CCR5 may drive migration of monocytes from PB to the tissues in humans and mice [11,12][16]. Control of monocyte release from the spleen in mice is rather specific because it is controlled by angiotensin II signaling [14].

Blood monocytes in various species consist of several subpopulations of cells differing in size, nuclear morphology, granularity, and functionality [17]. Based on the expression of cell surface molecules CD14, CD163 and SLA-DR, two major “steady-state” subpopulations of BM and PB monocytes have been described in pigs: CD14^hi^/CD163^-^/SLA-DR^-^ and CD14^low^/CD163^+^/SLA-DR^+^ monocytes [18]. During inflammation, the large subpopulation of “inflammatory” monocytes expressing very high levels of CD163, but probably lacking the SLA-DR molecule (thus being CD14^low^/CD163^+^/SLA-DR^-^), rapidly appear in the BM and PB and replace the CD14^low^/CD163^+^/SLA-DR^+^ subpopulation [19].

Although the pig serves as an important animal model for understanding innate human immunity [20], the current knowledge of monocyte migration into inflamed tissues is limited.

Actinobacillus pleuropneumoniae (APP) is the causative agent inducing pleuropneumonia in pigs. The bacteria bind to cells of the lower respiratory tract [21]. Clinical signs and pathological changes of the disease already appear within a few hours after experimental infection [22]. The infection is followed by the destruction of alveolar macrophages and a rapid influx of professional phagocytes and lymphocytes to the tissue and bronchoalveolar space [22]. A rapid cellular influx of MP into infected lungs together with a specific localization of the pathogen in the lungs predetermine experimental APP infection to be an appropriate model for observing MP migration under inflammatory conditions in pigs. It was previously demonstrated that porcine monocytes migrate into the lungs during inflammation induced by *Actinobacillus pleuropneumoniae*[19] where they become a source of pro-inflammatory cytokines (our unpublished data) and after a few days they probably develop into macrophages [19]. The role of chemokines and their receptors in monocyte trafficking in pigs is completely unknown.

The present study reveals that CD163^+^ monocytes infiltrate various tissues during inflammation. Moreover, changes in mRNA levels of chemokines and chemokine receptors during this process are demonstrated.

## Material and methods

### Animals

Fifteen eight-week-old healthy pigs from a herd with no history of APP infection or vaccination were used in the experiment. The pigs were kept in the accredited barrier-type animal facilities of the Veterinary Research Institute, Brno. The animal care protocol for this experiment followed the Czech guidelines for animal experimentation and was approved by the Branch Commission for Animal Welfare of the Ministry of Agriculture of the Czech Republic (permission No MZe 1223). The pigs were allowed to acclimatize in the animal facilities for two weeks before the experimental infection was performed.

Nine pigs were exposed to APP infection for 18 h and six pigs served as non-infected controls. The infection with an APP field strain (biotype 1, serotype 9, KL2-2000) from the fourth passage was performed intranasally during inhalation, administering 5 × 10^8^ bacteria suspended in phosphate buffered saline to the second third of each nasal cavity. Health status was monitored during the entire experiment and clinical signs of respiratory disorders were recorded (dyspnoea, cough, anorexia, depression and lethargy).

Euthanasia was performed by exsanguination after combined anesthesia with a TKX (Telazol-Ketamin-Xylazin) mixture containing 12.5 mg/mL tiletamine and 12.5 mg/mL zolazepam (Telazol, Virbac, Carros, France), 12.5 mg/mL ketamine (Vetoquinol, Lure, France) and 12.5 mg/mL xylazine (Bioveta, Ivanovice na Hane, Czech Republic), administered intramuscularly in a final volume of 0.2 mL/kg body weight.

### Blood and tissue sampling and processing

Heparinized peripheral blood (PB) samples in a total volume of 250 mL per pig were collected during euthanasia into 50 mL polypropylene tubes (TPP, Trasadingen, Switzerland). Moreover, BM, spleen, lung, tracheobronchial lymph node (TBLN) and mesenteric lymph node (MLN) samples were taken immediately after euthanasia. Samples were taken from the lungs of infected animals from three different sites as described previously [23]: (1) necrotic area (NA), (2) demarcation zone (DZ), and (3) visually unaffected area (UA). The samples were used for isolation of leukocytes that subsequently underwent flow cytometric analysis (BM, PB, spleen, lungs, TBLN, MLN) and cell sorting (BM, PB). A part of the tissue samples was frozen in liquid nitrogen and stored at -80 °C until further use for immunohistochemical staining. A part of tissue samples was placed into RNAlater, left at 4 °C overnight and then stored at -80 °C until further processing for RT-qPCR analysis.

The suspension of BM leukocytes was acquired as follows: the sternum bones were separated from the connective tissue, weighed and then BM leukocytes were collected by washing the bone tissue with pyrogen-free DPBS (Dulbecco’s Phosphate Buffered Saline, Sigma-Aldrich, St. Louis, USA). The suspension of splenic, lung (NA, DZ and UA), TBLN and MLN leukocytes was acquired as follows: a piece of the tissue was weighed, cut into small pieces (less than 1 mm^3^) and then the pieces were disintegrated by passing through a fine nylon mesh. Finally, the isolated BM, splenic, lung, TBLN and MLN leukocytes were washed with DPBS (centrifugation speed 626 *g*).

Then, concentration of leukocytes per mL of PB was assessed using an auto hematology analyzer (BC-2800Vet, Shenzhen Mindray Bio-Medical Electronics, Shenzhen, People’s Republic of China). Moreover, the total counts of the isolated BM, splenic, lung, TBLN and MLN leukocytes were assessed using the auto hematology analyzer and, together with the known original weight of the tissue from which the cells were isolated, the total leukocyte count per g of the tissue was calculated.

### Detection of cell surface molecules by flow cytometry

Staining of cell surface molecules was performed as described previously [18], with small modifications. Samples of BM, splenic, lung, TBLN or MLN leukocytes were lysed with ammonium chloride solution in order to remove contaminating red blood cells, washed, pelleted and stained with a cocktail of the following anti-CD antibodies: anti-CD203a (clone PM 18–7, IgG1 subclass, Serotec, Oxford, UK), anti-SLA-DR (clone MSA3, IgG2a subclass, VMRD, Pullman, USA), anti-CD14 (clone MIL-2, IgG2b subclass, Serotec, Oxford, UK), anti-SWC8 (clone MIL-3, IgM class, a generous gift from Dr J.K. Lunney, Animal Parasitology Institute, Beltsville, MO, USA) in the presence of heat-inactivated goat serum. Then, staining with a cocktail containing the following secondary antibodies was performed: anti-mouse IgG1: PE (Southern Biotech, Birmingham, USA), anti-mouse IgG2a: PE-Cy5.5 (Invitrogen, Carlsbad, USA), anti-mouse IgG2b: SPRD (Southern Biotech, Birmingham, USA) and anti-IgM: DyLight405 (Jackson ImmunoResearch Laboratories, West Grove, USA). Then, incubation with heat inactivated mouse serum was performed. After that, samples were stained with anti-CD172a (clone DH59B, IgG1 subclass, VMRD, Pullman, USA) pre-labeled with Zenon mouse-IgG1: Alexa Fluor 647 reagent (Invitrogen, Carlsbad, USA) and with anti-CD163 (clone 2A10/11, IgG1 subclass, Serotec, Oxford, UK) pre-labeled with Zenon mouse-IgG1: Alexa Fluor 488 (Invitrogen, Carlsbad, USA) and with propidium iodide (PI, Sigma-Aldrich, St. Louis, USA). The measurement was performed by the BD LSRFortessa cell analyzer (Becton-Dickinson, San Jose, USA). Three hundred thousand events were acquired. The post-acquisition analysis of data was performed using BD FACSDiva software (Becton-Dickinson, San Jose, USA).

**Figure 1:**
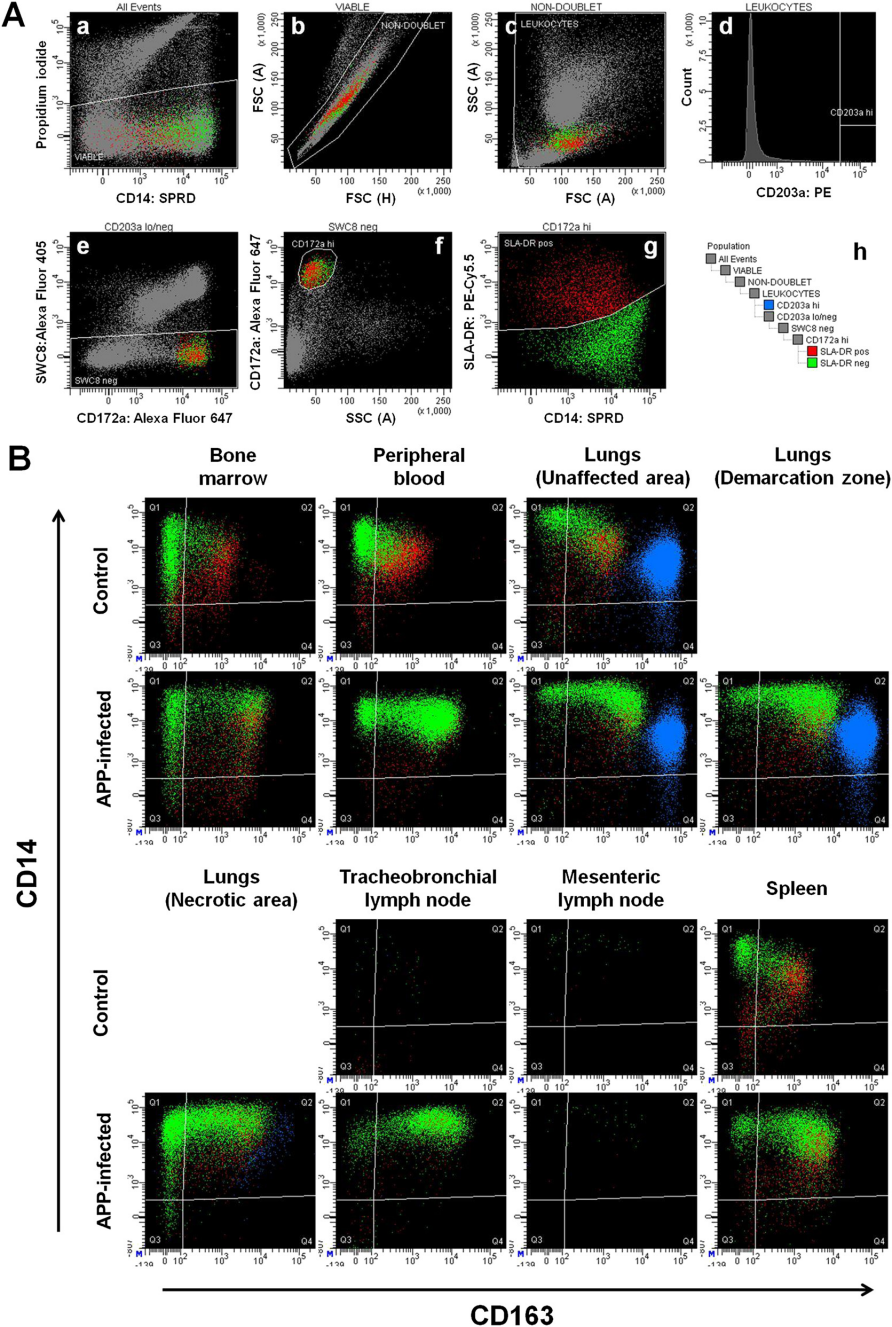
**Overview of flow cytometry analyses. (A)** Gating strategy for identification of monocytes and macrophages. The peripheral blood is shown. Designation of population shown within each dot-plot is indicated above the dot-plot. Leukocytes were identified as viable **(a)** non-doublet **(b)** cells with typical light scatter properties of leukocytes **(c)**. Then, macrophages were gated simply as CD203a^hi^ leukocytes **(d)** and marked with blue color. Monocytes were gated as CD203a^low/-^ SWC8^-^**(e)** CD172a^hi^**(f)** leukocytes where the CD203a^low/-^ region was defined as the complementary region to the CD203a^hi^ region. Then, SLA-DR^+^ monocytes were marked with red color and SLA-DR^-^ monocytes were marked with green color **(g)**. SLA-DR^-^ region was defined as the complementary region to the SLA-DR^+^ region. Gating order is shown in the scheme **(h)**. **(B)** Representative CD163 vs. CD14 dot-plots of macrophages (blue) and monocyte subpopulations (green: SLA-DR^–^, red: SLA-DR^+^) in various body compartments of control and APP-infected pigs.

The monocytes and macrophages were identified as described previously [19]. The general gating strategy is shown in Figure 1A. Then, two major monocyte subpopulations were identified according to CD14 and CD163 expression as CD14^hi^/CD163^-^ and CD14^low^/CD163^+^ monocytes. The total counts of monocytes in PB and various tissues were calculated using previously determined total leukocyte count per mL of PB or per g of the tissue. Moreover, intensity (median of fluorescence intensity, MFI) of CD163, CD14 and SLA-DR expression was ascertained in CD14^low^/CD163^+^ monocytes.

#### *Immunohistochemistry*

Frozen tissue samples were cut using a cryostat (CM1900, Leica Microsystems GmbH, Wetzlar, Germany), placed on Vectabond-covered slides (Vector Laboratories, Burlingame, USA), fixed with acetone at -20 °C, dried, and stored at -20 °C until subsequent staining. The endogenous peroxidase was quenched with Dual Endogenous Enzyme-Blocking Reagent (DAKO, Glostrup, Denmark) for 10 min. Slides were washed and the Protein Block (DAKO, Glostrup, Denmark) was applied for 5 min. Then, anti-CD163 antibody was applied and the slides were incubated for 60 min at 37 °C in a humid chamber. Then, the slides were washed and EnVision reagent (HRP, Mouse, DAKO) was added. The slides were incubated for 60 min at 37 °C in a humid chamber. The slides were washed and stained with DAB + (Liquid, DAKO, Glostrup, Denmark) for approx. 1–3 min. Then, the slides were washed with distilled water, lightly counterstained with Harris hematoxylin and mounted in glycerol-gelatin.

### Cell sorting

The CD163^+^ monocytes from APP-infected and control pigs were sorted. BM and PB mononuclear cells, freshly isolated from BM and PB samples by Lympholyte-H gradient (Cedarlane, Burlington, Canada) centrifugation at 800 *g*, were labeled with anti-CD163 antibody for 15 min at 4 °C. Then, they were thoroughly rinsed and incubated with Anti-Mouse IgG Micro Beads (Miltenyi Biotec, Bergisch Gladbach, Germany) for 20 min at 4 °C. The labeled cells were passed through MACS LS Separation Columns using Quadro Macs Separator (Miltenyi Biotec, Bergisch Gladbach, Germany) and CD163^+^ monocytes were collected. The purity of the collected CD163^+^ monocytes was evaluated by flow cytometry and was found to be higher than 95%. The cells were pelleted immediately after sorting, lysed with RLT buffer (Qiagen, Hilden, Germany) and stored at -80 °C until processing.

### Quantitative real-time PCR

The isolation of total RNA and quantitative real-time PCR analysis was performed as described previously [24]. Primers for the genes were designed using the freely available Primer3 software (Table 1) [25]. The annealing temperature was 58 °C. The specificity of real-time PCR was evaluated by analysis of Tm. Expression of each gene was calculated as a multiple of the reference gene expression using the following formula: [1/(E^CtGene^)]/ [1/(E^CtReference Gene^)] where E represents the amplification efficiency of the primer pair used. Amplification efficiency was higher than 1.95 for each gene. The data are expressed in relation to the reference gene Hypoxanthine-guaninephosphoribosyltransferase (HPRT) [26].

**Table 1:** Primers for chemokines, chemokine receptors, CD62L and reference gene used in quantitative real-time PCR.

**Gene**	**Alternative name**	**Primer forward / reverse**	**Tm**	**Length of product**
CCL1	I-309	CCGTGGTTCTGGTGTGCCTGCTG / ACATGCATGCTCTTGCTGTCCACCT	68.19/67.01	70
CCL2	MCP-1	CCGAAGCTTGAATCCTCATC / TAGCAGCAGGTGACTGGAGA	56.30/60.25	134
CCL3-like1	MIP-1αS	TTTTGAGACCAGCAGCCAGT / TCAGCTCCAGGTCAGAGATG	59.82/58.52	122
CCL4	MIP-1β	GCTCCCAGCCAGCCGTGGTATTC / CAGTCATCACTGGGGTTGGCGCA	67.64/67.50	70
CCL5	RANTES	ACCACACCCTGCTGTTTTTC / GGCGGTTCTTTCTGGTGATA	58.89/57.60	124
CCL8	MCP-2	CAGTGTCCCCAGGAGGCTGTGATCTTC / GTCCGCGCAGACCTCCTTGTCG	68.18/67.74	60
CCL11	Eotaxin	TCCCCAGAATGCTGTGATCTTCAGCAC / GGTCCAGGTACTCCATGGCATTCTGAACC	66.79/68.35	98
CCL17	TARC	CTCCTCCTGGGGGCTTCCCTGC / CAGCACTCCCGGCCCACGTTG	68.64/68.37	68
CCL19	ELC	CCACGCTGAAAGGTCGCGAGCTC / TTGCGGTGGTGCTTGCTCTTAGC	68.18/66.13	106
CCL20	MIP-3α	TGCAGCAAGTCAGAAGCAGCAAGC / CGAGCTGCTGTGTGAAGCCCATG	66.60/66.44	100
CCL21	SLC	CCCCCACACCCAAGGCAGTGATG / AACCCAGGCTTGGCTCCTGCTTC	67.60/67.31	116
CCL22	MDC	TCAGACTCCTGCCGGAGGCCTG / CAGGGCAGTCTGGGGTCAGCAC	67.96/67.30	80
CCL25	TECK	TTCTTCCAAGGCCCTCAGTCTGGAGTGA / ACTGTGGGCTCATGGTCCTGGAATAGC	68.56/68.05	147
CCL27	CTACK	CCAGGCCTTCGTGCTTCACCTGTC / TCCCCTGGAGCCTCCTTCCATGG	67.69/67.54	104
CCL28	MEC	GAAGCTATACTTCCCATTGCCTCCAGCT / TGCGCTTGACATGAAGGATGACAGCA	66.27/67.17	148
CXCL9	Mig	AGCAGTGTTGCCTTGCTTTTGGGTATCATC / GCTGGTGTTGATGCAGGAACAACGTCC	67.76/68.40	102
CXCL10	IP-10	CCCACATGTTGAGATCATTGC / CATCCTTATCAGTAGTGCCG	57.57/55.44	168
CXCL11	I-TAC	ACAAAACAGAAGTGATTGTCACCCTGAAAGCAC / GCTTGCTTTGATTTGGGATTTAGGCATCTTCGT	67.68/67.90	73
CXCL12	SDF-1α/β	CCTCGTGCTGACCGCGCTCTG / CGAGAGAGTGGGACTGGGTTTGTTTAAAGCT	68.06/68.07	256
CXCL14	BRAK	CGGCCAGCATGAGGCTCCTGAC / ACTTGCATTTGGACCCGTCCACGC	67.66/68.16	87
CXCL16	SR-PSOX	TCGCGGAGAATGTGGACGTGCTC / TCGTCTGGGCAGGGGTGCTACTG	67.20/68.13	128
CX3CL1	Fractalkine-like	GGCTGCTGGCCTTCCTTGGTCTC / GGGAGCAGCCCTGCAGACTCTGG	67.59/68.97	78
CCR1		GCTCCAGAAACAAAGACTTCGTGGACACAG / ATGTTGCTGCTAAAGGCAGTGGGCT	67.58/67.26	70
CCR2		TGCCACTTGGAAGAAGCAACAGACCG / CAGAGAGTGAGATGTGGGCAGCACG	67.54/67.21	100
CCR3		TCCCCATCAACGGGAGAGCAGGA / CTTCAGAGCTTCAGTGCTGTGCTGG	67.16/65.93	137
CCR4		GCCATTGTGCACGCGGTCTTCTC / GTGAAGCGAACACAGCCACCGAC	66.91/66.55	97
CCR5		TGGTCAGAGGAGCTGAGACA / AGAAGGGACTCGTCGTTTGA	59.89/58.67	86
CCR6		AGCGAGAGGGCGCTGGGCTAC / GTTGAGGCAGCAGTGCAGGAACG	68.88/66.60	69
CCR7		GTGGTGGCTCTCCTTGTCAT / GAAGCACACGGACTCGTACA	59.67/60.04	114
CCR9		CCACAGAAGCCGCAAGTCTGATGC / TGGCTTGCAAACTGCCTGACATGGT	66.45/67.64	139
CCR10		CTGCAGCTGCCCTACAGTCTCGC / AGATCCTTGCGCTTGCTGGCAGG	67.54/67.85	95
CXCR3		GTAGGGTGGACGTAGCCAAG / GGAACTTGACACCCACGAAG	59.82/58.77	96
CXCR4		TACCATGGACGGGTTCCGTATATTCACTTCAG / GCATTTTCCTCCCGGAAACAGGGTTCC	67.26/67.89	108
CXCR6		CCACTGACAGAGCACCTCTACTGGGTC / CCCCTGGCTGCTGTCGTTGGAAG	67.67/67.77	106
CX3CR1		CCAGTCACCTGCTGGCAACCCTACC / GGAAGCTGGTTTTGGGCTCTGGCTC	68.98/67.96	88
CD62L		AGCTTCTTGTCAGCCCAGGTCATGC / CCCAGAGGCTCACACTGAGTCACGAAC	67.25/68.46	132
CD163		CTTGGGGCAGCGTTGGCAGGAATAG / ATGCAGGGCTGATGTCCCCTCTGTC	68.02/68.16	99
SLA-DR		TGCTTGGAGTGTCACCATCTG / GGTCTGCTCTTTGTTGCTGTG	60.27/60.00	115
TBP1		AACAGTTCAGTAGTTATGAGCCAGA / AGATGTTCTCAAACGCTTCG	59.75/56.48	153
HPRT		GAGCTACTGTAATGACCAGTCAACG / CCAGTGTCAATTATATCTTCAACAATCAA	61.41/58.88	111

### Statistical analysis

Statistical evaluation of data was performed by the Mann–Whitney U test. The differences in CD163, CD14 and SLA-DR molecule expression in various organs was evaluated by the Kruskal-Wallis test and Dunn’s post-test (GraphPad Prism, Version 3.02, GraphPad Software, San Diego, USA).

## Results

### Distribution and phenotypic characterization of CD14^hi^/CD163^-^ and CD14^low^/CD163^+^ monocytes from various organs

The appearance of CD14^hi^/CD163^-^ and CD14^low^/CD163^+^ monocytes was evaluated in BM, PB, spleen, lungs, TBLN and MLN from control and APP-infected pigs by flow cytometry.

The monocytes from control pigs were found in BM, PB, spleen and lungs (Figure 1B). No monocytes were found in TBLN and MLN. In conformity with our previous study [18], CD14^hi^/CD163^-^ monocytes were almost exclusively SLA-DR^-^ while CD14^low^/CD163^+^ monocytes were mostly SLA-DR^+^.

After APP infection, monocytes were still present in BM, PB, spleen and UA of the lungs. Moreover, they appeared in DZ as well as in NA of lungs. Although they massively infiltrated TBLN, they did not appear in MLN, suggesting that monocytes appeared specifically in the LN, which drain the inflamed tissue. The CD14^low^/CD163^+^ monocytes from infected pigs were almost exclusively SLA-DR^-^ and show much higher expression of CD163 on the contrary to CD14^low^/CD163^+^ monocytes from control pigs and consistently with our previous findings [19]. Because the CD14^low^/CD163^+^ monocytes created during APP-induced inflammation differed markedly from CD14^low^/CD163^+^ monocytes in control pigs, we hereinafter call them “inflammatory” and “steady-state” monocytes, respectively.

Then, total counts of monocyte subpopulations and lung macrophages per mL of PB or per g of tissue were calculated. The numbers of CD14^hi^/CD163^-^ monocytes in BM, PB and UA of the lungs and spleen decreased after infection and they were not elevated in either organ except a small population in TBLN (Table 2). On the contrary, the numbers of CD14^low^/CD163^+^ monocytes were elevated in PB and the UA of the lungs and, moreover, monocytes massively appeared in TBLN. On the contrary, their counts did not change significantly in BM, DZ and NA of the lungs and in the spleen.

**Table 2:** Monocyte subpopulations in various body compartments from control and APP-infected pigs.

			**CD163**^**-**^		**CD163**^**+**^		**% CD163**^**+**^	
		**n**	**Control**	**APP-infected**	**Control**	**APP-infected**	**Control**	**APP-infected**
**Bone marrow**		5	1571 ± 118	464 ± 83**	1020 ± 161	1430 ± 142	38.6 ± 2.7	75.2 ± 4.3**
**Peripheral blood**		5	418 ± 61	184 ± 44*	418 ± 111	1142 ± 255*	47.1 ± 6.9	86.2 ± 0.5**
**Spleen**		4	3009 ± 916	1052 ± 273*	3524 ± 1,043	10 886 ± 3359	54.8 ± 9.6	90.0 ± 1.8*
**Lungs**	**Unaffected area**	5	1459 ± 336	735 ± 225*	3384 ± 702	7555 ± 1493*	69.0 ± 7.4	90.9 ± 2.1**
	**Demarcation zone**	4		985 ± 467		11 890 ± 4331		90.4 ± 3.8*
	**Necrotic area**	5		2873 ± 1,441		5948 ± 2232		66.2 ± 6.0
**Tracheobronchial LN**		5	n.d.	1547 ± 674*	n.d.	16 563 ± 4284*	n.d.	92.9 ± 1.7
**Mesenteric LN**		4	n.d.	n.d.	n.d.	n.d.	n.d.	n.d.

The data are shown as total count × 10^3^ of monocyte subpopulations per g of tissue of per mL of PB or as a percentage of CD163^+^ monocytes from CD163^+^ and CD163^-^ monocytes as measured by flow cytometry. Data are expressed as mean ± S.E.M. The significant differences (Mann–Whitney U test) between control and APP-infected pigs are marked with *P < 0.05, **P < 0.01. The values which were elevated between control and APP-infected pigs are highlighted in bold. *LN*, lymph node, *n.d*, non-detected.

The counts of lung macrophages were not changed significantly in UA and DZ of lungs from APP-infected pigs when compared to controls. On the contrary, lung macrophages almost completely disappeared in the NA of the lungs (Figure 1B) and their counts in NA were significantly decreased (*P* < 0.01) compared to controls (UA: 10 385 ± 2724; DZ: 21 335 ± 10 587; NA: 603 ± 1376; control 18 708 ± 3454 expressed as 10^3^ macrophages per g of lung tissue). Moreover, the macrophages in NA resembled the CD163 and CD14 expression pattern of monocytes in NA rather than that of macrophages in UA and DZ of the lungs (Figure 1B) suggesting, in accordance with our previous data [19], that the macrophages in NA of lungs developed from recruited monocytes after the original population of pulmonary macrophages completely disappeared.

### Localization of CD14^low^/CD163^+^ monocytes within tracheobronchial lymph nodes, spleen and mesenteric lymph nodes

**Figure 2:**
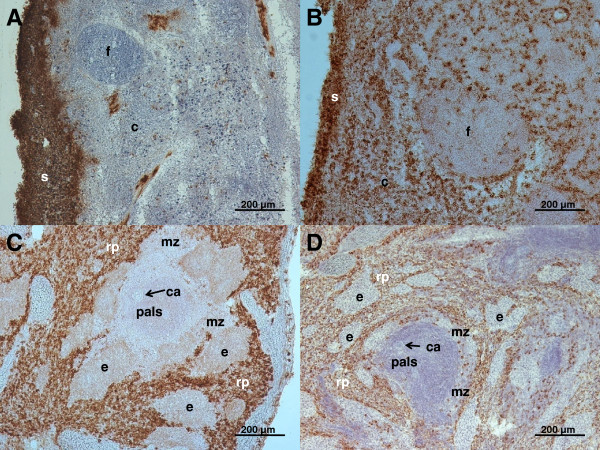
**Representative pictures of immunohistochemical detection of CD163**^**+ **^**cells in tracheobronchial lymph node and spleen.** CD163^+^ cells were detected in tracheobronchial lymph nodes from control **(A)** and APP-infected pigs **(B)** and in spleen from control **(C)** and APP-infected pigs **(D)**. Immunohistochemical visualization: horseradish peroxidase, brown substrate, hematoxylin counterstain; c, cortex; ca, central artery; e, ellipsoid; f, follicle; mz, marginal zone; pals, periarterial lymphatic sheath, rp, red pulp; s, subcapsular sinus.

In order to ascertain localization of CD14^low^/CD163^+^ monocytes within TBLN and the spleen, immunohistochemical staining of the CD163 molecule in these tissues was performed. Although no monocytes were detected in TBLN of control pigs by flow cytometry, the immunohistochemistry revealed numerous CD163^+^ cells within the subcapsular sinus of TBLN (Figure 2A). These cells were relatively large with abundant cytoplasm and expressed the CD172a molecule (data not shown) suggesting that they represent subcapsular sinus macrophages. The discrepancy between data from flow cytometry and immunohistochemistry can be explained by the fact that the LN leukocytes for flow cytometry analysis were isolated by simple disintegration of the tissue. Therefore, it is probable that macrophages were not harvested by this procedure because they are tightly fixed in the tissue. When immunohistochemical staining of the CD163 molecule in TBLN from APP-infected pigs was compared with the control ones, it was revealed that abundant CD163^+^ cells massively infiltrated the cortex and paracortical zone of TBLN (Figure 2B). These cells were also found within the follicles but rarely.

Immunohistochemical staining of the spleen show no differences in localization of CD163^+^ cells between control and APP-infected pigs. The occurrence of CD163^+^ cells was limited to the red pulp and marginal zone only (Figure 2C, D). CD163^+^ cells in the marginal zone were present as single events and probably represented marginal zone macrophages rather than monocytes.

Immunohistochemical staining of MLN revealed that there were, similarly to TBLN from control pigs, CD163^+^ cells within the MLN simus in both control and APP-infected pigs, which represent macrophages of the subcapsular sinus. According to flow cytometry results, no monocytes within cortex and paracortex were found in control or APP-infected pigs (data not shown).

### Intensity of CD163, CD14 and SLA-DR molecule expression by CD14^low^/CD163^+^ monocytes in various organs

**Figure 3:**
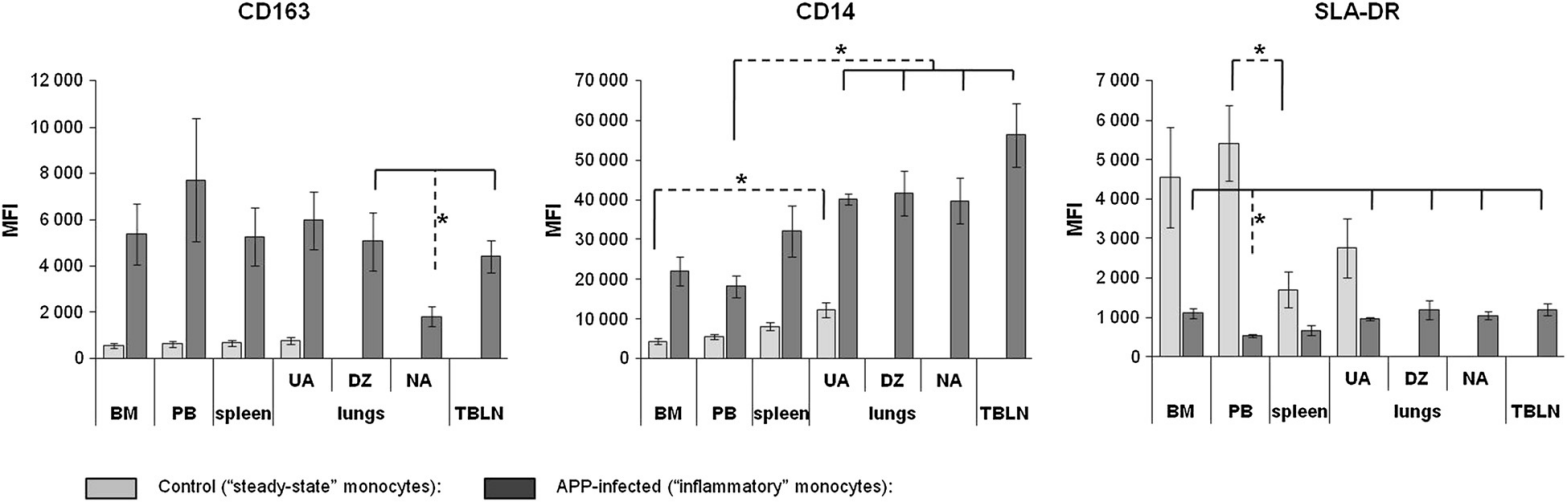
**Intensity of CD163, CD14 and SLA-DR expression by CD14**^**+**^**/CD163**^**+ **^**monocytes in various body compartments.** Data are shown as MFI (Median of fluorescence intensity) ± S.E.M. BM, bone marrow (*n* = 5); PB, peripheral blood (*n* = 5), UA, lungs-unaffected area (*n* = 5), DZ, lungs-demarcation zone (*n* = 4), NA, lungs-necrotic area (*n* = 5), TBLN, tracheobronchial lymph node (*n* = 5); MLN, mesenteric lymph node (*n* = 4), spleen (*n* = 4). The significant differences (Kruskal-Wallis test) amongst particular compartments are indicated.

In order to reveal potential relationships among CD14^low^/CD163^+^ monocytes in various tissues, the MFI of CD163, CD14 and SLA-DR molecule expression was evaluated in CD14^low^/CD163^+^ monocytes from BM, PB, spleen, lungs and TBLN of control and APP-infected pigs (Figure 3).

The MFI of CD163, CD14 and SLA-DR sharply differed between control and APP-infected pigs. As it was already apparent from Figure 1, the “inflammatory” CD14^low^/CD163^+^ monocytes from APP-infected pigs expressed significantly more CD163 and CD14 and less SLA-DR than their “steady-state” counterparts from control pigs. The MFI of SLA-DR expression in APP-infected pigs was very low and corresponded to the MFI of SLA-DR-negative cells.

On the contrary, what was not apparent from Figure 1 is that some differences in MFI of CD163, CD14 and SLA-DR in CD14^low^/CD163^+^ monocytes occurred, also among individual organs (see Kruskal-Wallis test in Figure 3). The order of organs in Figure 3 was created according to the known ways of monocyte migration through particular organs in the mouse model. As summarized in the introduction, monocytes leave the BM and after circulation in PB they settle in the spleen. From PB or alternatively from the spleen, they migrate to the inflamed tissues. Then, from the inflamed tissues or alternatively from PB they enter the LN.

The expression of CD163 was unchanged in monocytes from control as well as from APP-infected pigs with the exception of NA of the lungs where CD163 expression was down-regulated.

On the contrary, CD14 expression continuously increased dependently on the supposed way of monocyte apearance within organs, suggesting that porcine monocytes could follow the same way of migration as mouse monocytes do. This increase was more pronounced in “inflammatory” monocytes. The CD14 MFI of spleen “inflammatory” monocytes was on the half-way between the PB and lungs. We can speculate that, as in the mouse, PB monocytes could come into the spleen before they enter the inflamed tissue. Moreover, MFI of CD14 in “inflammatory” monocytes from TBLN was significantly elevated compared to PB, while no significant difference was observed between TBLN and the lungs, suggesting that monocytes that infiltrate the TBLN are rather recruited from inflamed lungs than from PB.

SLA-DR expression by “steady-state” monocytes was sharply decreased between PB and the spleen, and PB and the lungs. On the contrary, the expression was comparable between the spleen and lungs. We can speculate that “steady-state” lung monocytes could be recruited rather from the spleen than from PB. However, it may also reflect a down-regulation of SLA-DR expression after appearance of monocytes in the tissues, independently of whether or not they go to the tissues from PB through the spleen.

### Chemokine and chemokine receptor mRNA expression in various organs

In order to elucidate which chemokines and their appropriate receptors could play a role in monocyte distribution during inflammation, the mRNA levels for all porcine chemokines and their receptors, for which the cDNA sequence is available, were measured in control and APP-infected pigs. The mRNA level of porcine chemokines was measured in BM, lungs, TBLN, spleen and MLN.

It was found that the mRNA for CCL3, CCL8 and CCL19 was significantly up-regulated in BM from APP-infected pigs (Table 3). Many of the evaluated chemokines were elevated to high levels in NA compared to UA and DZ of the lungs. On the contrary, only a few of them, namely CCL3, CCL4 and CCL19 or CCL3, CCL19 and CXCL14 were found to be slightly elevated in UA or DZ of lungs, respectively (Table 3). In TBLN, the CCL3, CCL4, CCL19, CXCL14 and CXCL16 were elevated (Table 3). It should be added that, especially in TBLN, many chemokines were down-regulated in APP-infected pigs. Namely CCL17, CCL21, CCL25, CCL28, CXCL9, CXCL10, CXCL11 and CXCL12 mRNA were decreased suggesting that these chemokines may play a homeostatic role during the steady-state conditions in pigs. Surprisingly, although “inflammatory” monocytes were detected in the spleen of APP-infected pigs, none of the measured chemokines was found to be increased in the spleen (data not shown). Neither chemokine was found to be increased in the MLN; this was in accordance with the fact that monocytes were not found in MLN from APP-infected pigs. The only exception was CXCL14, which was slightly, but significantly up-regulated (data not shown). Generally, the levels of chemokine mRNA in MLN resembled the levels in TBLN from control pigs.

**Table 3:** Chemokine expression in various organs from control and APP-infected pigs.

	**Bone marrow**						**Lungs**												**Tracheobronchial LN**					
	**Control**			**APP-infected**			**Control**			**APP-infected**									**Control**			**APP-infected**		
										**Unaffected area**			**Demarcation zone**			**Necrotic area**								
CCL1	0.028	±	0.018	0.050	±	0.037	0.025	±	0.006	0.013	±	0.002	0.008	±	0.002*	**0.152**	**±**	0.055*	0.009	±	0.001	0.007	±	0.001
CCL2	0.195	±	0.043	0.486	±	0.183	1.799	±	0.291	2.027	±	0.394	2.785	±	0.831	**101.809**	**±**	25.084**	3.831	±	0.228	5.296	±	1.162
CCL3	0.117	±	0.015	**0.174**	**±**	0.026*	0.623	±	0.121	**2.149**	**±**	0.497**	**1.521**	**±**	0.313*	**148.299**	**±**	48.339**	0.240	±	0.057	**1.169**	**±**	0.307**
CCL4	0.103	±	0.017	0.148	±	0.024	0.805	±	0.108	**2.006**	**±**	0.451*	1.506	±	0.458	**57.529**	**±**	15.897**	0.291	±	0.057	**0.922**	**±**	0.158**
CCL5	0.015	±	0.014	0.054	±	0.000	2.610	±	0.455	2.015	±	0.322	1.259	±	0.210	**11.351**	**±**	1.899**	2.293	±	0.481	1.540	±	0.179
CCL8	0.099	±	0.013	**0.158**	**±**	0.024*	0.121	±	0.023	0.110	±	0.016	0.125	±	0.012	0.428	±	0.153	0.336	±	0.080	0.380	±	0.038
CCL11	0.003	±	0.001	0.001	±	0.000	0.002	±	0.001	0.004	±	0.002	0.007	±	0.003	**0.132**	**±**	0.032**	0.002	±	0.001	0.006	±	0.004
CCL17	0.000	±	0.000	0.001	±	0.000	0.033	±	0.012	0.025	±	0.006	0.021	±	0.005	0.120	±	0.031	0.715	±	0.085	0.430	±	0.080*
CCL19	0.183	±	0.039	**1.478**	**±**	0.690**	1.526	±	0.097	**3.689**	**±**	0.540**	**3.813**	**±**	0.728*	**39.163**	**±**	9.875**	45.957	±	4.607	**77.840**	**±**	7.874*
CCL20	0.000	±	0.000	0.001	±	0.000	0.009	±	0.002	0.010	±	0.002	0.019	±	0.003	**6.833**	**±**	1.846**	0.288	±	0.046	0.736	±	0.217
CCL21	0.016	±	0.008	0.006	±	0.001	0.976	±	0.107	1.053	±	0.166	1.147	±	0.189	0.533	±	0.138*	17.522	±	2.483	6.464	±	2.130**
CCL22	0.003	±	0.001	0.002	±	0.001	0.038	±	0.013	0.024	±	0.006	0.024	±	0.004	**0.153**	**±**	0.040*	0.673	±	0.124	0.306	±	0.049*
CCL25	0.007	±	0.001	0.006	±	0.001	0.013	±	0.001	0.011	±	0.001	0.013	±	0.001	0.011	±	0.001	0.010	±	0.001	0.006	±	0.001**
CCL27	0.000	±	0.000	0.001	±	0.000	0.002	±	0.000	0.003	±	0.000	0.002	±	0.000	0.005	±	0.002	0.001	±	0.000	0.001	±	0.000
CCL28	n.d.			n.d.			0.036	±	0.003	0.038	±	0.013	0.045	±	0.032	0.011	±	0.003**	n.d.			n.d.		
CXCL9	0.180	±	0.026	0.141	±	0.018	1.704	±	0.180	1.186	±	0.288	0.820	±	0.197*	1.644	±	0.740	4.188	±	0.591	1.790	±	0.225**
CXCL10	0.983	±	0.222	0.996	±	0.197	5.272	±	0.425	3.685	±	0.742	2.788	±	0.545*	8.892	±	4.605	6.644	±	0.826	3.097	±	0.531**
CXCL11	0.353	±	0.209	0.036	±	0.013	0.124	±	0.037	0.072	±	0.023	0.059	±	0.017	0.457	±	0.201	0.146	±	0.038	0.030	±	0.007**
CXCL12	1.061	±	0.123	1.022	±	0.224	0.378	±	0.047	0.241	±	0.034*	0.246	±	0.040	0.453	±	0.081	6.123	±	0.842	2.024	±	0.580**
CXCL14	0.794	±	0.075	1.376	±	0.322	1.935	±	0.241	2.553	±	0.381	**4.312**	**±**	0.547*	**35.412**	**±**	4.900**	2.961	±	0.400	**5.579**	**±**	0.586**
CXCL16	0.605	±	0.058	0.372	±	0.036*	3.371	±	0.211	2.340	±	0.106**	2.639	±	0.343*	**9.151**	**±**	0.881**	1.427	±	0.092	**2.510**	**±**	0.486**
CX3CL1	0.010	±	0.004	0.025	±	0.006	0.078	±	0.017	0.044	±	0.011	0.050	±	0.010	**0.639**	**±**	0.225*	0.106	±	0.008	0.048	±	0.016*

The data are shown as a fold change of chemokine mRNA expression in relation to the reference gene HPRT as measured by the RT-qPCR. Data are expressed as mean ± S.E.M. (Control: *n* = 6, APP-infected: *n* = 9). The significant differences (Mann–Whitney U test) between control and APP-infected pigs are marked with *P < 0.05, **P < 0.01. The values which were elevated between control and APP-infected pigs are highlighted in bold. *LN*, lymph node, *n.d*, non-detected.

It should be highlighted that the levels of CCL19 mRNA in TBLN and MLN from control pigs were naturally high compared to other tissues, reaching more that 10-times higher levels. These high levels were not, however, accompanied by the appearance of any monocytes in TBLN and MLN of control pigs suggesting that CCL19 probably could not play a role at all in attracting monocytes into LN.

It should be further highlighted that CXCL14 was up-regulated in MLN from APP-infected pigs but this up-regulation was also not accompanied by the appearance of any monocytes. Although the timing of the infection could influence the appearance of monocytes in MLN, this calls into question the role of CXCL14 in attracting “inflammatory” monocytes into LN.

Various chemokines were found to be elevated in BM, lungs and TBLN of APP-infected pigs. Accordingly, the principle of chemokine-mediated attraction of cells into the tissues is based on interactions of chemokines with chemokine receptors expressed by the cells; the level of mRNA for chemokine receptors was evaluated in BM and PB CD163^+^ monocytes, in NA of lungs and in TBLN.

BM and PB CD163^+^ monocytes expressed relatively high levels of CCR1, CCR3, CCR4, CCR10, CXCR3, CXCR4 and CX3CR1 (Table 4) compared to CCR2, CCR5 and CCR7, which were expressed in relatively lower levels. Other chemokine receptors (CCR6, CCR9, and CXCR6) were undetectable or expressed at negligible levels. Generally, if any differences were found in chemokine receptor mRNA expression between BM and PB monocytes, the expression in PB was always up-regulated. The main expectation was that chemokine receptors, which are involved in chemotaxis of monocytes into tissues during inflammation, should be up-regulated in APP-infected pigs and, on the contrary, chemokine receptors with homeostatic function should be down-regulated. Surprisingly, if any differences were found in chemokine receptor expression between control and APP-infected pigs, the expression in APP-infected pigs was always down-regulated. Due to this uniform down-regulation of most chemokine receptors, the stability of the HPRT gene was proven by measurement of another commonly used reference gene TBP1. No significant differences in TBP1 expression in relation to HPRT were observed (Table 4) suggesting that HPRT is stable throughout all BM and PB CD163^+^ monocytes from control and APP-infected pigs and the observed down-regulation of most of the chemokine receptors is correct.

**Table 4:** **Chemokine receptor, CD62L, CD163 and TBP1 expression by bone marrow and peripheral blood CD163**^**+ **^**monocytes from control and APP-infected pigs.**

	**Bone marrow**						**Peripheral blood**					
	**Control**			**APP-infected**			**Control**			**APP-infected**		
**CCR1**	0.87	±	0.12 *	0.47	±	0.07 *	0.87	±	0.13	1.09	±	0.30
**CCR2**	0.35	±	0.07 *	0.18	±	0.02 *	0.23	±	0.08	0.11	±	0.03
**CCR3**	4.42	±	1.00 *†	1.86	±	0.62 *‡	18.79	±	4.77 †	9.21	±	4.28 ‡
**CCR4**	3.24	±	0.71 *†	1.33	±	0.41 *‡	13.44	±	3.25 †	6.42	±	3.00 ‡
**CCR5**	0.16	±	0.03 *	0.06	±	0.01 *‡	0.30	±	0.05	0.15	±	0.05 ‡
**CCR6**	n.d.			n.d.			n.d.			n.d.		
**CCR7**	0.46	±	0.12 †	0.26	±	0.05	0.88	±	0.17 ^0^†	0.24	±	0.10 ^0^
**CCR9**	0.003	±	0.001	0.001	±	0.000	0.006	±	0.002 ^0^	0.001	±	0.000 ^0^
**CCR10**	2.62	±	0.54 *†	1.09	±	0.31 *‡	10.54	±	2.26 ^0^†	4.86	±	2.00 ^0^‡
**CXCR3**	2.55	±	0.55 *†	1.00	±	0.29 *‡	10.83	±	2.58 †	5.10	±	2.27 ‡
**CXCR4**	3.81	±	0.38 *†	2.04	±	0.15 *‡	8.70	±	0.68 †	9.05	±	1.82 ‡
**CXCR6**	0.002	±	0.001 *†	0.000	±	0.000 *	0.019	±	0.008 ^0^†	0.001	±	0.000 ^0^
**CX3CR1**	2.96	±	0.63 †	1.18	±	0.31 ‡	13.01	±	3.05 †	6.33	±	2.33 ‡
**CD62L**	0.81	±	0.20	0.86	±	0.21	0.77	±	0.32	1.29	±	0.27
**CD163**	5.31	±	0.52 *†	**9.08**	**±**	2.27 *‡	11.98	±	2.55 †	**19.40**	**±**	**2.43 ‡**
**SLA-DR**	16.62	±	2.77 *†	4.43	±	0.76 *	40.60	±	4.04 ^0^†	4.23	±	1.23 ^0^
**TBP1**	0.06	±	0.01	0.06	±	0.01	0.09	±	0.02	0.11	±	0.02

The data are shown as a fold change of chemokine receptor, CD62L, CD163, SLA-DR and TBP1 reference gene mRNA expression in relation to the reference gene HPRT as measured by the RT-qPCR. Data are expressed as mean ± S.E.M. (*n* = 5). The significant differences (Mann-Whitey U test, *P* < 0.05,) between control and APP-infected pigs are marked with * (bone marrow) or ^0^ (peripheral blood). The significant differences (Mann–Whitney U test, *P* < 0.05) between bone marrow and peripheral blood monocytes are marked with † (control) or ‡ (APP-infected). The values which were elevated between control and APP-infected pigs are highlighted in bold. *N.d*, non-detected.

The flow cytometry shows that CD163^+^ monocytes from APP-infected pigs expressed more CD163 and less SLA-DR protein compared to control pigs. Comparable data were observed at mRNA level in PB monocytes and in the tissues that had been infiltrated by these cells (i.e. NA of lungs and TBLN, Table 4) verifying, at least partly, that the data obtained at the mRNA level resemble the expression at the protein level.

In the NA of the lungs, CCR1, CCR3, CCR4, CCR10 and CXCR4 were up-regulated in APP-infected pigs compared to the lungs from control pigs (Table 5). In TBLN, CCR1, CCR2, CCR3 and CXCR4 were up-regulated in the APP-infected pigs compared to control pigs (Table 5).

**Table 5:** Chemokine receptor, CD62L and CD163 expression in lungs from control and APP-infected pigs.

	**Lungs**						**Tracheobronchial LN**					
	**Control**			**APP-infected (necrotic)**			**Control**			**APP-infected**		
**CCR1**	0.260	±	0.021	**5.462**	**±**	1.152**	0.091	±	0.014	**1.930**	**±**	0.673**
**CCR2**	0.070	±	0.009	0.087	±	0.015	0.076	±	0.008	**0.174**	**±**	0.029*
**CCR3**	0.163	±	0.018	**2.574**	**±**	0.594**	0.198	±	0.055	**0.738**	**±**	0.158**
**CCR4**	0.054	±	0.013	**0.494**	**±**	0.180*	0.333	±	0.058	0.244	±	0.055
**CCR5**	0.189	±	0.016	0.324	±	0.071	0.085	±	0.007	0.175	±	0.052
**CCR6**	n.d.			n.d.			n.d.			n.d.		
**CCR7**	0.055	±	0.010	0.094	±	0.021	1.748	±	0.215	1.386	±	0.293
**CCR9**	0.051	±	0.007	0.030	±	0.004*	0.181	±	0.014	0.130	±	0.015
**CCR10**	0.021	±	0.002	**1.205**	**±**	0.411**	0.015	±	0.002	0.021	±	0.003
**CXCR3**	0.206	±	0.034	0.719	±	0.255	0.550	±	0.044	0.290	±	0.047**
**CXCR4**	0.792	±	0.132	**1.798**	**±**	0.296**	0.825	±	0.052	**2.628**	**±**	0.328**
**CXCR6**	0.157	±	0.029	0.047	±	0.011**	0.100	±	0.013	0.083	±	0.015
**CX3CR1**	0.338	±	0.038	0.704	±	0.252	0.115	±	0.012	0.101	±	0.017
**CD62L**	0.83	±	0.08	**35.55**	**±**	9.82**	3.75	±	0.202	**13.21**	**±**	2.618**
**CD163**	1.54	±	0.11	**5.85**	**±**	1.20**	1.67	±	0.507	**14.81**	**±**	2.561**

The data are shown as a fold change of chemokine receptor, CD62L and CD163 gene mRNA expression in relation to the reference gene HPRT as measured by the RT-qPCR. Data are expressed as mean ± S.E.M. (Control: *n* = 6, APP-infected: *n* = 9). The significant differences (Mann–Whitney U test) between control and APP-infected pigs are marked with *P < 0.05, **P < 0.01. The values which were elevated between control and APP-infected pigs are highlighted in bold. *LN*, lymph node; *n.d*, non-detected.

**Figure 4:**
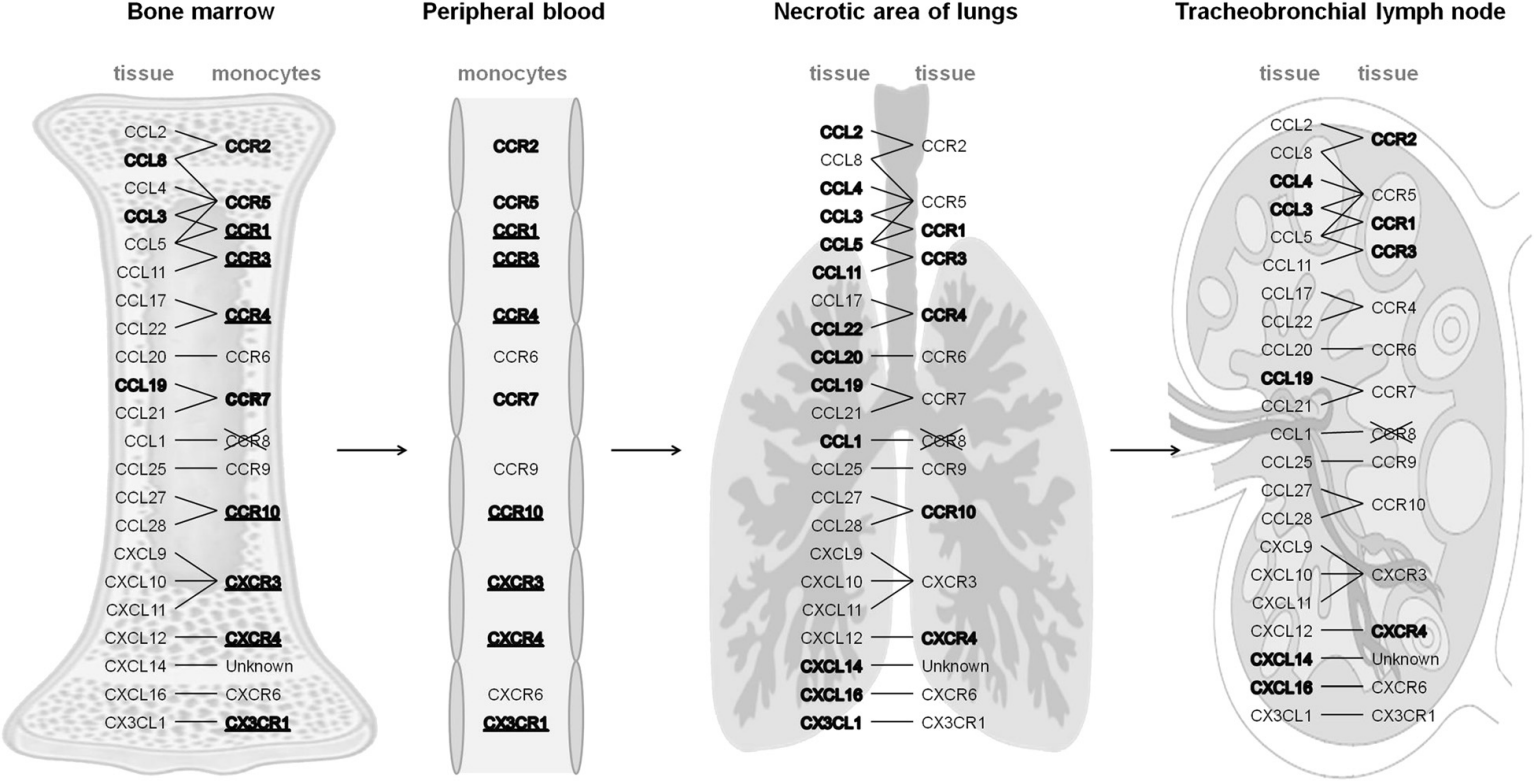
**Scheme of chemokines and corresponding chemokine receptor mRNA expression in various organs and CD163**^**+ **^**monocytes.** Chemokines in bone marrow, necrotic area of the lungs and tracheobronchial lymph nodes and chemokine receptors in necrotic areas of the lungs and tracheobronchial lymph nodes which were up-regulated in the APP-infected pigs compared to control are highlighted in bold. Chemokine receptors in bone marrow and peripheral blood CD163^+^ monocytes which were expressed at relatively lower levels are highlighted in bold. Those expressed in relatively high levels are highlighted in bold and underlined. Arrangement of the scheme was created based on the review of Bonecchi et al. [27].

To obtain a comprehensive overview, we have drawn a chart that summarizes the results of chemokines and chemokine receptor mRNA expression in various organs and in CD163^+^ BM and PB monocytes (Figure 4).

Beside chemokines, monocyte recruitment into inflamed tissues could also be controlled by the adhesion molecule CD62L [13]. Therefore, CD62L mRNA expression was measured in BM and PB CD163^+^ monocytes, in the lungs and in TBLN from control and APP-infected pigs. Uniformly low levels of CD62L mRNA were found to be expressed by CD163^+^ monocytes (Table 4). On the contrary, CD62L expression was strongly elevated in the NA of lungs and in TBLN from APP-infected pigs (Table 5) suggesting that CD62L molecule could play a role in control of cell appearance in the inflamed tissue and LN. However, it is not clear whether or not this principle is utilized just by “inflammatory” monocytes, since they expressed only low levels of CD62L mRNA when they were present in the PB.

## Discussion

The present study shows that monocyte counts in various organs changed during inflammation. Our previous study revealed that monocytes apeared in the inflamed lungs during APP-induced pleuropneumonia [19]. The present study extends our knowledge about the distribution of monocytes during inflammation. In accordance with our previous data the CD163^+^ monocyte counts were found to be significantly increased in PB, UA of lungs and in TBLN from APP-infected pigs suggesting that monocytes migrate just to these organs. On the contrary, the numbers of these monocytes did not change in BM, DZ and NA of the lungs and in the spleen. The fact that no change occurred in the BM monocyte count can be simply explained by enhanced emigration of newly generated BM monocytes into other organs as described previously [19]. However, the question arises as to whether monocytes migrated to DZ and NA of the lungs and into the spleen during APP infection when their counts were unchanged. The analysis of CD163, CD14 and SLA-DR expression shows that CD163^+^ monocytes from APP-infected pigs shared a relatively uniform phenotype in all evaluated organs. This phenotype was completely different from the phenotype of CD163^+^ monocytes from control pigs. This suggests that original “steady-state” monocytes disappeared from tissues and were replaced with a novel population, which could be called “inflammatory” and which is characterized by a high CD163 expression, increased CD14 expression and an almost non-occurring SLA-DR expression. Therefore, it is probable that monocytes were not found to be elevated in DZ and NA of the lungs and in the spleen because the loss of the “steady-state” and appearance of “inflammatory” monocytes was in balance.

The possibility that “inflammatory” CD14^low^ CD163^+^ monocytes arise from “steady-state” CD14^low^ CD163^+^ or CD14^hi^ CD163^-^ monocytes was also taken into account during evaluation of the data. We have no direct evidence that this does not occur, however, there was a uniform phenotypic profile of “inflammatory” monocytes as well as relatively high numbers of “inflammatory” monocytes which strongly exceeded the numbers of those “steady-state” ones.

In the extensively used mouse model, one specific subset of monocytes, which is characterized by high levels of Ly-6C molecule expression, is released from PB [16,28] or from the spleen [9] to the site of acute inflammation. In mice, there is no known information concerning the phenotype of this subset which somehow changes between the steady-state and inflammatory conditions. From this point of view, the pig is completely different because the CD14^low^/CD163^+^ “steady-state” monocytes are replaced by the phenotypically distinct “inflammatory” subset during APP infection.

Levels of mRNA for various chemokines and chemokine receptors were found to be changed in lymphoid tissue, lungs and monocytes. Although we cannot exclude that mRNA expression for some chemokine receptors can be turned down to a basal level after entry of monocytes into the tissues in our time-point, i.e. 18 h after infection, only chemokine receptors with up-regulated mRNA expression were considered to play a role in recruitment of monocytes. Thus the following strategy for a biological interpretation of the data was suggested: if expression of a chemokine in an inflamed tissue was found to be up-regulated and the chemokine plays a role in attracting monocytes to the tissue, it could be assumed that at least one of the appropriate chemokine receptors should be expressed by these monocytes. After monocytes had appeared in the tissue, the mRNA for this/these chemokine receptor(s) should be up-regulated in the tissue. The last presumption was supported by the evaluation of CD163 mRNA expression in PB monocytes and in the tissue which was infiltrated by these cells (i.e. NA of lungs and TBLN). As described above, “inflammatory” monocytes from PB expressed high levels of CD163 mRNA. Accordingly, CD163 mRNA was strongly elevated in the NA of the lungs and in TBLN.

The present study indicates that, after APP infection, “inflammatory” monocytes originating in BM appeared in various organs. Since chemokines that are responsible for monocyte emigration are produced directly in the BM [10], we evaluated chemokines expressed in BM and appropriate chemokine receptors expressed by CD163^+^ BM monocytes. The CCL3, CCL8 and CCL19 were up-regulated in BM of APP-infected pigs. BM monocytes expressed chemokine receptors appropriate to these chemokines, although it should be noted that the levels of expression were relatively lower. Therefore, we can hypothesize that any of these chemokines could possibly initiate a release of BM monocytes to the bloodstream. In the mouse model, the CCL2 expression in the BM and CCL2/CCL7-CCR2 signaling plays a fundamental role in monocyte emigration from the BM under inflammatory conditions [10,15]. Our results suggest that CCL3, CCL8 or CCL19 could be responsible for this effect, while CCL2 is not, because it was not up-regulated in BM from APP-infected pigs, on the contrary to the findings in the mouse model. The finding closely correlates with the fact that porcine and mouse monocyte subsets, which participate in the acute phase of inflammatory response in each of these species, feature different CCR2 expression. In the mouse, these Ly-6C^hi^ monocytes typically express high levels of CCR2 [17]. On the contrary, porcine “steady-state” as well as “inflammatory” CD163^+^ monocytes in control and APP-infected pigs express very low levels of CCR2 and, moreover, it is known that they are not attracted by CCL2 in vitro [29].

The present study shows that, as well as in the mouse [9], porcine spleen contained high numbers of monocytes. Moreover, similarly to the mouse, the proportion of particular spleen monocyte subpopulations resembled those in PB under steady-state conditions. In the mouse, the spleen functions as an important reservoir of monocytes [9,14]. During inflammation, monocytes are rapidly released from this niche to the inflamed tissues and the total number of splenic monocytes rapidly decreases by one half [14]. In pigs, the splenic “steady-state” CD163^+^ monocytes also disappeared from the spleen; however, they were substituted by “inflammatory” CD163^+^ monocytes. Based on the analysis of the intensity of CD14 and SLA-DR molecule expression, we can speculate that, similarly to mice, PB monocytes migrate to the spleen before they enter the target tissue. Further experiments, using in vivo tracking methods, are planned in the future to reveal the role of the spleen in monocyte trafficking. Similarly to the mouse [9], monocytes were localized in the red pulp of the spleen only. Since they were not found within the white pulp of the spleen in either control or APP-infected pigs, it could be supposed that they probably do not influence the specific immune response within the spleen by e.g. presenting the antigen to T cells, as described in LN draining the inflamed tissue [2,4].

Although the spleen is primarily a reservoir of monocytes in the mouse, in the case of certain specific diseases such as malaria, monocytes migrating to the spleen could play a role in the control of this disease [8]. Since this migration is modulated by certain chemokines [30] we evaluated chemokine expression in the spleen from APP-infected pigs. We found that although “inflammatory” monocytes appeared in the spleen in APP-infected pigs, neither of the evaluated chemokines was elevated suggesting that chemokines are probably not involved in monocyte migration to the spleen.

The proportions of particular monocyte subpopulations in the spleen resembled those in the PB from control or APP-infected pigs. Owing to the fact that the spleen is an organ which naturally contains high amounts of blood, it should therefore be discussed as to whether monocytes found in the spleen are not only an artifact. The total numbers of CD163^+^ monocytes shows that 1 g of splenic tissue contains almost ten-times the amount of monocytes in 1 mL of PB. Since 1 mL of PB weighs approximately 1 g, it can be concluded that the numbers of splenic monocytes greatly exceed their numbers in the PB and therefore, they are probably settled in the spleen and their presence is not an artifact. The exact role and fate of splenic monocytes in pigs is, however, unknown.

It was also demonstrated in the present study that “inflammatory” CD163^+^ monocytes appeared in the lung tissue after APP-infection. In NA of the lungs, different chemokines were up-regulated. However, CCL3, CCL5, CCL11 and CCL22 were the only chemokines whose at least one of appropriate receptor (CCR1, CCR3 or CCR4) was up-regulated. So, they could probably play a role in attracting cells to the inflamed tissue. As mentioned before, monocytes arrive at the site of inflammation from the PB or spleen. All the receptors, which were elevated in NA of the lungs (CCR1, CCR3, CCR4, CCR10 and CXCR4) were expressed at high levels also by PB monocytes, suggesting that any of the above mentioned chemokines could play a role in attracting monocytes to the NA of the lungs. CCR10 and CXCR4 were elevated in the NA of the lungs, although all known ligands for these chemokines (CCL27 and CCL28 or CXCL12, respectively) were not increased. This elevation was probably caused entirely by the fact that monocytes infiltrating the site of inflammation expressed these receptors, although they did not use them for chemotaxis. It is to be noted that although mRNA for chemokine receptors, expressed by infiltrating monocytes, probably influences the expression of the chemokine receptors CXCR3 and CX3CR1, which were also expressed at high levels by PB monocytes, was not elevated in NA of the lungs. A possible explanation is that monocytes attracted to the inflamed tissue down-regulate mRNA for CXCR3 and CX3CR1 when moving from PB to the inflamed tissue. Additionally, CCL1 and CXCL14 were also up-regulated in the NA of the lungs. Biological relevance of this feature cannot be further confirmed, because the cDNA sequence for CCR8, which is the receptor for CCL1, is not available and the receptor for CXCL14 is generally unknown.

The present study also shows that high numbers of monocytes appeared in TBLN after APP infection. Although various chemokines were up-regulated in TBLN, the only CCL3 with the up-regulated appropriate receptor CCR1 probably plays a role in attracting cells to the inflamed tissue. As mentioned above, the receptor for CXCL14 is generally unknown, and therefore, we cannot decide whether this receptor plays a role in monocyte migration or not. However, CXCL14 was up-regulated in MLN from APP-infected pigs but this up-regulation was not accompanied by the appearance of any monocytes. From this perspective, it seems less likely that CXCL14 played a role in attracting “inflammatory” monocytes into inflamed tissues.

It should be noted that CCR2, CCR3 and CXCR4 receptors were also up-regulated, but none of the measured ligands was elevated. However, a role of CCR2 and CCR3 in monocyte migration to the TBLN could not be excluded because these receptors bind a range of various other pro-inflammatory chemokines. These chemokines were unfortunately non-measurable due to the unavailability of cDNA sequences for these chemokines in pigs.

Migration of monocytes into LN in mice is driven by CCL19/CCL21-CCR7 signaling [4,7] or by CCL3, CCL4 and CXCL9 [12]. Our study shows that, unlike in mice, CCL3 and its appropriate receptor CCR1 are probably involved in monocyte attraction to the LN during inflammation in pigs. Although CCL4 and CCL19 were also found to be up-regulated in TBLN from APP-infected pigs, the corresponding receptors (CCR5 and CCR7 respectively) were unchanged in TBLN. Generally, high steady-state expression of CCL19 in the lymph node is known to be responsible for homeostatic lymphocyte migration [31]. Since high CCL19 expression in TBLN from control pigs was not accompanied by monocyte appearance, it could therefore be presumed that, in contrast to mice, CCL4 and CCL19 probably do not play a role in monocyte recruitment to LN draining the inflamed tissue.

It is not clear from the present study as to what is the origin of “inflammatory” TBLN monocytes in pigs. In mice, monocytes migrate to the LN from inflamed tissues via the lymphatic vessels [2,5,6] or from PB via high endothelial venules (HEV) [2,12]. Although TBLN were massively infiltrated with monocytes, the chemokine receptors which were elevated in TBLN (CCR1, CCR2, CCR3, CXCR4) did not correspond with the profile of chemokine receptors, which were highly expressed by “inflammatory” monocytes in PB (CCR1, CCR3, CCR4, CCR10, CXCR3, CXCR4 and CX3CR1). They rather resembled the chemokine receptors which were elevated in NA of the lungs infiltrated with “inflammatory” monocytes (CCR1, CCR3, CCR4, CCR10 and CXCR4). Based on the differences in the expression of chemokine receptors, we can speculate that “inflammatory” monocytes could arrive at TBLN from NA of the lungs rather than from PB.

Our study revealed that, similarly to the mouse [4,5], “inflammatory” monocytes accumulate in the cortex and paracortex of LN suggesting that they could play a role in the induction and regulation of T cell responses. However, the majority of studies in mice reported that monocytes, which populate the LN during inflammation, develop into monocyte-derived DC [4,13] exhibiting typical features of DC, such as high MHCII expression already 12 hours after infection [4]. Our study, on the contrary, revealed that “inflammatory” monocytes in TBLN as well as in other organs after 18 h-lasting APP infection exhibited uniformly low expression of a porcine MHCII antigen, the SLA-DR molecule. Therefore, it is possible that monocytes infiltrating the TBLN of APP-infected pigs could play a role since they are without development into DC and similarly to very recent findings in mice [7], they could be involved in cross-presentation of antigens to cytotoxic T cells.

It could be concluded that different chemokines and chemokine receptor mRNA expression was changed in various organs and cells during APP infection in pigs. Given together, only CCL3, CCL8 and CCL19 in BM, CCL1, CCL3, CCL5, CCL11, CCL22 and CXCL14 in the necrotic area of the lungs and CCL3 in TBLN could probably play a role in monocyte attracting into these organs. The present study shows for the first time that there are large differences in phenotypical changes and distribution of monocytes during inflammation between pigs and the widely used mouse model.

## Competing interests

The authors declare that they have no competing interests.

## Authors’ contributions

PO conceived the study, its design and coordination, wrote the manuscript, participated in collection and processing of the samples, and participated in the immunohistochemical, immunofluorescent, and flow cytometric analyses. LL sorted the cells and participated in collection and processing of the samples, and flow cytometric analysis. ZK prepared the APP cultures and performed the experimental infection. MV and MM performed RT-qPCR analysis. MF helped writing the final version of the manuscript. All authors read and approved the final manuscript.
